# A comprehensive list of genes required for the efficient conjugation of plasmid Rts1 was determined by systematic deletion analysis

**DOI:** 10.1093/dnares/dsae002

**Published:** 2024-02-01

**Authors:** Takahiro Murata, Yasuhiro Gotoh, Tetsuya Hayashi

**Affiliations:** Department of Pediatrics, Teikyo University School of Medicine, Mizonokuchi Hospital, Takatsu-ku, Kawasaki, Kanagawa 213-8507, Japan; Department of Bacteriology, Faculty of Medical Sciences, Kyushu University, Higashi-ku, Fukuoka 812-8582, Japan; Department of Bacteriology, Faculty of Medical Sciences, Kyushu University, Higashi-ku, Fukuoka 812-8582, Japan

**Keywords:** Rts1 plasmid, conjugation, deletion analysis, mobilization assay, integrating and conjugative element

## Abstract

While conjugation-related genes have been identified in many plasmids by genome sequencing, functional analyses have not yet been performed in most cases, and a full set of conjugation genes has been identified for only a few plasmids. Rts1, a prototype IncT plasmid, is a conjugative plasmid that was originally isolated from *Proteus vulgaris*. Here, we conducted a systematic deletion analysis of Rts1 to fully understand its conjugation system. Through this analysis along with complementation assays, we identified 32 genes that are required for the efficient conjugation of Rts1 from *Escherichia coli* to *E. coli*. In addition, the functions of the 28 genes were determined or predicted; 21 were involved in mating-pair formation, three were involved in DNA transfer and replication, including a relaxase gene belonging to the MOB_H12_ family, one was involved in coupling, and three were involved in transcriptional regulation. Among the functionally well-analysed conjugation systems, most of the 28 genes showed the highest similarity to those of the SXT element, which is an integrative conjugative element of *Vibrio cholerae*. The Rts1 conjugation gene set included all 23 genes required for the SXT system. Two groups of plasmids with conjugation systems nearly identical or very similar to that of Rts1 were also identified.

## 1. Introduction

Bacterial conjugation plays important roles in bacterial evolution and adaptation to the environment, including the acquisition and dissemination of antimicrobial resistance (AMR) and/or bacterial virulence.^1–3^ Conjugation is mediated by two types of mobile genetic elements (MGEs), conjugative plasmids and integrative conjugative elements (ICEs), the latter of which cannot replicate autonomously but are integrated into host chromosomes in a site-specific manner and can be excised from chromosomes to form circular molecules for conjugation.^4^

Many genes are necessary to drive this complex biological system, including those required for pilus formation, mating pair stabilization, channel formation, conjugative DNA metabolism, and gene expression control.^5–7^ The conjugation system is one of the three functional types of type IV secretion systems (T4SSs), with the other two types being involved in DNA uptake from or release into the extracellular milieu and the injection of proteins called effectors into host cells or other bacterial cells.^8–11^ Thus, conjugation genes include a set of genes for the T4SS.

The process of assembling the channel complex and sex pili in conjugation is called mating-pair formation (Mpf).^5,12,13^ The origin of transfer (*oriT*) is recognized and cleaved by a relaxase. Some auxiliary proteins are also involved in DNA processing; they form a relaxosome along with the relaxase and transferable DNA molecules.^12^ This process is called DNA transfer and replication (Dtr). The relaxosome and the channel complex are connected by a type IV coupling protein (T4CP). Since relaxases and one of the Mpf proteins, which are VirB4 homologues, are widely conserved and they are the major components for Dtr and Mpf, respectively, conjugation systems are phylogenetically divided into nine or eight groups based on their sequences, respectively.^13–15^ With an increasing amount of sequence information on conjugative elements from a wide range of bacterial species, gene sets possibly responsible for conjugation functions have been identified based on sequence homologies. However, functional analyses have not yet been performed for most cases. Therefore, the full set of genes required for conjugation has been experimentally defined for only a very limited number of prototype or representative plasmids/ICEs.^16–19^

Rts1 is a self-transmissible kanamycin (Km)-resistance plasmid that is 217,182 bp in length and was originally found in a clinical isolate of *Proteus vulgaris*.^20^ Rts1 is a prototype of the T-incompatibility group^21^ and expresses pleiotropic thermosensitive phenotypes in autonomous replication,^22^ conjugative transfer,^20^ host cell growth,^23^ and the restriction^13,14^ of T-even phages.^24^ We previously determined the whole-genome sequence of Rts1.^25^ Although the host range of Rts1 has not yet been systematically analysed, it has been shown that Rts1 can be transferred by conjugation from *P. vulgaris* belonging to the family *Morganellaceae* which was recently created in the order *Enterobacterales*^26^ to *Escherichia coli* and *Salmonella enterica*, both of which belong to the family *Enterobactericeae* in the order *Enterobacterales*.^20^ Our previous homology search of 300 open reading frames (ORFs) on the Rts1 genome identified a set of genes that may be related to conjugation, and many of them showed the noticeable sequence similarity to those of the conjugation systems of plasmids F or R27, which are prototypes of IncFI and IncHI1 plasmids, respectively.^25^ However, the functions of these genes were not analysed. In this study, to fully understand the conjugation system of Rts1 and identify all of the required components, we conducted a systematic plasmid-wide deletion analysis of Rts1.

## 2. Materials and methods

### 2.1. Bacterial strains, plasmids, and media

*Escherichia coli* BW25113 (delta(*araD-araB*)*567*, delta(*rhaD-rhaB*)*568*, delta *lacZ4787* (::rrnB-3), and *hsdR514*, *rph-1*)^27^ was used as the host strain of Rts1 at the gene deletion step and the donor strain in the conjugation assay. *Escherichia coli* HB101 (*leuB6, supE44, thi-1, hsdS20, recA13, ara-14, proAB, lacY1, galK2, rpsL20, xyl-5,* and *ntl-1*) was used as the recipient strain in the conjugation assays. The plasmids pKD46, pKD3, and pCP20 were used for gene deletion.^27^ Plasmids used for the complementation assay were selected from a set of pUC18 derivatives that were previously prepared for random shotgun sequencing of Rts1,^25^ and pACYC184 was also used as a cloning vector for the complementation assay for three genes (see the complementation assay section for details). The plasmid pSU4628 (CloDF13::Tn*A* ∆ *Eco*RV) carrying the ampicillin (Ap)-resistance gene was used for the mobilization assay.^28^ All strains were cultivated in lysogeny broth (LB) or 2xYT media supplemented with appropriate antibiotics, including Ap at 100 or 200 µg ml^−1^, Km at 30 µg ml^−1^, streptomycin (Sm) at 200 µg ml^−1^, and chloramphenicol (Cm) at 30 µg ml^−1^. Rts1 DNA was purified as described previously.^25^ Routine DNA manipulation and the preparation of genomic and plasmid DNA were carried out by standard methods. DNA sequencing was performed by ABI377 (Applied Biosystems). *Escherichia coli* strain ER1648 which contains Rts1^25^ is available in the Prokaryotes (*E. coli*) program in the National BioResource Project (https://nbrp.jp/en/resource/prokaryote-en/).

### 2.2. Deletion of genes or gene blocks from the Rts1 genome

Deletion of a gene or a block of genes was introduced into the Rts1 genome by the one-step gene inactivation method,^27^ in which the lambda Red recombinase from pKD46 was utilized to facilitate the recombination of linear PCR products into the plasmid genome. pKD3 was used as a template for PCR, and the PCR products were introduced into cells by electroporation using GenePulsar II (Bio-Rad). The PCR products contained the Cm-resistance gene with 50-70 bp tails identical to the sequences that flank the region targeted for deletion. In addition, FLP recognition target sites were introduced on both sides of the Cm-resistance gene to eliminate the resistance gene by the FLP recombinase provided from pCP20. Thus, the gene (or gene block)-deletion mutants that were constructed were resistance marker gene-free and principally avoided polar effects introduced by gene insertion.^27^ The primers used in this step are listed in Supplementary Table S1. Each deletion was confirmed by the product size of PCR using a pair of outside primers that were used for deletion.

### 2.3. Conjugation and mobilization assays

*Escherichia coli* BW25113 carrying Rts1 or its mutants (Km-resistant) and HB101 (Sm-resistant) were used as the donor and recipient, respectively. The *E. coli* cells were cultivated overnight in a 2xYT medium containing appropriate antibiotics at 37°C. Donor and recipient cells were transferred to antibiotic-free fresh 2xYT medium with 100- and 10-fold dilutions, respectively, and the cells were incubated for 3 h at 30°C without shaking. Then, 10 µl of donor cells and 100 µl of recipient cells were mixed with 900 µl of antibiotic-free 2xYT medium, incubated for 90 min at 30°C without shaking, and spread on LB plates containing Km and Sm with appropriate dilutions. The transfer frequency of Rts1 (or its mutants) was calculated as the number of transconjugants per donor cell. For the mobilization assay using pSU4628, pSU4628 (encoding the Ap resistance gene) was introduced into BW25113 cells carrying Rts1 or its mutants, which then served as donor cells. The donor and recipient cells were cultivated, mixed, incubated as described above, and spread on LB plates containing Ap and Sm. The transfer frequency of pSU4628 was calculated as the number of pSU4628 transconjugants per donor cell.

### 2.4. Complementation assay

Plasmid clones used for the complementation assay were selected from the pUC18-based random shotgun library of Rts1 used for genome sequence determination^25^ based on the end sequences of each insert so that the expression of inserted genes was under the control of the pUC18-encoded *lac* promoter. pACYC184 served as a cloning vector for three ORFs, Orf191, Orf223, and Orf224, as the pUC18-based clone carrying Orf191 caused adverse effects in terms of host cell growth, and appropriate pUC18-based clones carrying Orf223 and Orf224 were not found in the library. For the cloning of these ORFs into pACYC184, each PCR-amplified DNA fragment was inserted into the *Bam*HI site of pACYC184. The PCR primers used are listed in Supplementary Table S2.

For the complementation assay, these plasmid clones were introduced into donor cells containing a relevant Rts1 mutant by electroporation, and the conjugation assay was performed as described above, except that Ap- or Cm-containing 2xYT medium was used for the cultivation of donor cells carrying pUC18- and pACYC184-derived plasmids, respectively. To avoid overexpression of the inserted genes, isopropyl β-D-thiogalactopyranoside (IPTG) was not added to the culture media during the conjugation assay. The introduction of the empty pUC18 or pACYC184 vector did not alter the conjugation efficiency of Rts1.

### 2.5. Homology search and replicon typing

For sequence homology and domain searches of the ORFs of Rts1, BLASTP (https://www.ncbi.nlm.nih.gov/blast.cgi/) and InterPro (https://www.ebi.ac.uk/interpro/) were used, respectively. To identify conjugation systems similar to that of Rts1, the NCBI database was searched by TBLASTN ver 2.14.1 using the Orf201 and Orf180 protein sequences as queries. GenomeMatcher v3.06^29^ was used for the sequence comparison of Rts1 with other plasmids and visualization of the results. PlasmidFinder v2.0.1^30^ was used for replicon typing.

## 3. Results

### 3.1. Overview and re-evaluation of conjugation-related genes in Rts1

**Figure 1.**
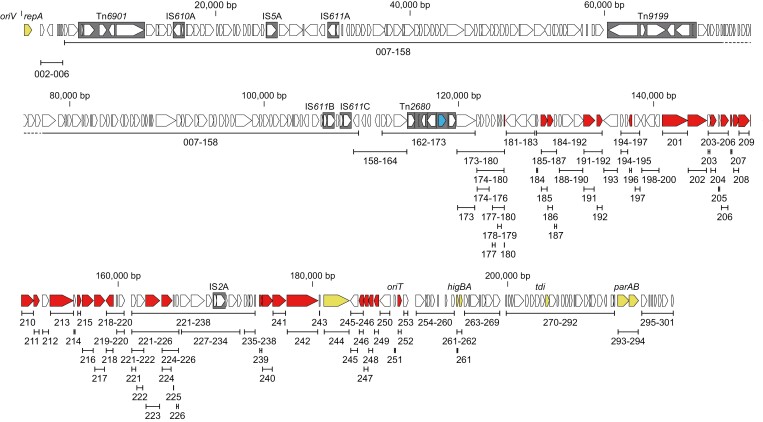
The gene organization of Rts1 and deletion mutants constructed in this study. The numbers under each line indicate the deleted ORF(s). Conjugation-related genes identified in this study are shown in red, and those for plasmid replication and maintenance are shown in yellow. The Km-resistance gene carried by Tn*2680* is indicated in blue.

**Figure 2.**
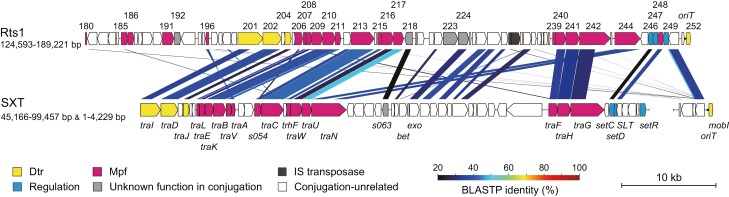
Comparison of the conjugation-related regions of Rts1 and SXT. The gene organizations of the conjugation-related regions of Rts1 with SXT and amino acid sequence homologies between genes are shown. Note that *traI* is located next to *mobI* in the SXT element.

We re-performed a homology search of all predicted 300 ORFs of Rts1 (Fig. 1) against the genes of functionally well-characterized conjugation elements and found homologs not only in plasmids F and R27 but also SXT, a 100-kb multidrug resistance-encoding ICE originally discovered in *V. cholerae* O139^31,32^ whose conjugation system has also been functionally characterized^16,18,19,31,33,34^ (Table 1, see Supplementary Table S3 for the revised annotation data of SXT). Of the conjugation systems of the three elements, the ORFs of Rts1 showed the highest similarity to those of SXT. Consistent with this result, the conjugation systems of Rts1 and SXT belong to the MOB_H12_ family, whereas those of R27 and F belong to the MOB_H11_ and MOB_F12_ families, respectively.^35^ The MPF types of Rts1, SXT and R27 were F type (MFP_F_).^36^ The gene organization was also relatively well conserved between Rts1 and SXT (Fig. 2). We found that 19 ORFs of Rts1 (Orf196, Orf201, Orf202, Orf204, Orf207-Orf211, Orf213, Orf215-Orf218, Orf240-Orf242, Orf244, and Orf246) exhibited noticeable amino acid sequence similarity to the gene products required for the conjugation of SXT^19,31,33^ (Table 1). Although Orf185 and Orf252 did not show noticeable homology to any gene products of SXT, the former showed noticeable homology to TrhP of R27, and the latter contained the MobI domain, suggesting its role in DNA transfer. Two ORFs (Orf224 and Orf248) exhibited noticeable homology to s066 and s082 of SXT, respectively, but these gene products were reported as not required for the conjugation of SXT.^19^ Orf180 and Orf206 also exhibited sequence similarities to TrbE of F and R0135 of R27, respectively, but TrbE was not required for the conjugation of F,^37^ and the function of R0135 is unknown.^18,34^ Thus, in this analysis, 21 ORFs (the 19 ORFs plus Orf185 and Orf252; indicated by asterisks in Table 1) were identified as potentially related to the conjugation of Rts1. These genes were located in a 60.8-kb region (the region from Orf185 to Orf252 in Fig. 1). The 455-bp region containing *oriT*, which we experimentally identified in our previous study,^25^ was located between *orf251* and *orf252*.

**Table 1.** The 32 genes required for the efficient conjugation of Rts1 from *E. coli* to *E. coli*

Orf number^a^	Gene name	Length (a.a.)	Conjugation efficiency^b^	Conjugation efficiency after complementation^b, c^	Mobilization efficiency of pSU4628^b^	Predicted function	Notes	Homologs: % identity (% coverage)		
								SXT (MOB_H12_)	R27 (IncHI1, MOB_H11_)	F (IncFI, MOB_F12_)
180	*orf180*	58	<0.0001	0.14	<0.0001	Mpf				trbE; 22.86% (60%)^e^
185*	*orf185(lepB)*	241	0.00097	0.046	0.0002	Mpf	LepB domain		TrhP; 25% (47%)	
186	*orf186*	214	<0.0001	2.6	<0.0001	Mpf				
191	*orf191*	410	0.056	0.51	0.031	Mpf				
192	*orf192*	221	0.018	0.94	1.2					
196*	*traA*	112	<0.0001	0.11	<0.0001	Mpf (pilin)	TrbC/VIRB2 family	TraA; 36.36% (88%)		
201*	*traI*	892	<0.0001	NT	3.1	Dtr (relaxase)	TraI_2 domain	TraI; 33.9% (44%)		
202*	*traD*	655	<0.0001	NT	1.43	coupling protein	SXT_TraD domain	TraD; 37.86% (94%)	TraG; 25.59% (79%)	
204*	*traJ*	219	<0.0001	0.21	2.8	Dtr (auxiliary protein)	DUF4400 domain	TraJ; 25.4% (86%)	TraJ; 22.92% (62%)	
206	*orf206(dsbC)*	281	0.0058	0.61	0.0011	Mpf	DsbG domain		R0135; 22.87% (74%)^f^	
207*	*traL*	94	<0.0001	NT	<0.0001	Mpf	TraL domain	TraL; 35.48% (98%)		
208*	*traE*	205	<0.0001	NT	<0.0001	Mpf	TraE domain	TraE; 31.61% (75%)	TrhE; 22.47% (85%)	
209*	*traK*	388	<0.0001	NT	<0.0001	Mpf	TraK domain	TraK; 35.65% (64%)	TrhK; 26.59% (65%)	
210*	*traB*	452	<0.0001	NT	<0.0001	Mpf	TraB domain	TraB; 38.88% (98%)	TrhB; 39.47% (50%)	traB; 27.27% (56%)
211*	*traV*	196	<0.0001	NT	<0.0001	Mpf	TraV domain	TraV; 42.41% (80%)	TrhV; 35.48% (59%)	traV; 23.43% (74%)
213*	*traC*	828	<0.0001	NT	<0.0001	Mpf	TraC-F-type domain	TraC; 42.63% (98%)	TrhC; 25.55% (96%)	traC; 24.82% (97%)
215*	*trhF*	150	<0.0001	0.76	<0.0001	Mpf	TraF domain (peptidase_S26 domain)	TrhF; 38.96% (100%)	TrhP; 33.33% (82%)	
216*	*traW*	416	<0.0001	NT	<0.0001	Mpf	TrbC_F type domain/TraW domain^d^	TraW; 26.25% (70%)	TrhW; 26.75% (55%)	TraW; 25.96% (45%)
217*	*traU*	358	<0.0001	NT	<0.0001	Mpf	TraU domain	TraU; 49.4% (92%)	TrhU; 32.26% (84%)	TraU; 32.68% (84%)
218*	*orf218*	271	0.015	0.18	0.96			s063; 20.6% (73%)		
223	*orf223(bet)*	510	0.013	0.031	1.3		RecT domain			
224	*orf224(exo)*	375	0.014	0.1	0.85		phage_rel_nuc domain	exo; 30.2% (78%)^e^		
239	*orf239*	108	<0.0001	0.12	<0.0001	Mpf				
240*	*traF*	363	<0.0001	NT	<0.0001	Mpf	TraF domain (pfam13728)	TraF; 33.66% (85%)	TrhF; 27.83% (84%)	TraF; 24.7% (68%)
241*	*raH*	483	<0.0001	NT	<0.0001	Mpf	TraH domain	TraH; 32.42% (95%)	TrhH; 29.3% (54%)	TraH; 29.39% (62%)
242*	*traG*	1098	<0.0001	NT	<0.0001	Mpf	TraG_N domain	TraG; 29.93% (49%)		TraG; 23.08% (43%)
244*	*traN*	900	<0.0001	NT	<0.0001	Mpf	TraN domain	TraN; 37.01% (97%)	TrhN; 24.32% (80%)	
246*	*setC*	163	<0.0001	1489	<0.0001	regulation (positive)	FlhC domain	SetC; 20.81% (90%)		
247	*setD*	174	<0.0001	97	<0.0001	regulation (positive)				
248	*orf248(slt)*	186	0.00025	0.12	0.0001	Mpf	SLT domain	s082; 35.29% (90%)^e^		
249	*setR*	193	402	9.2	137	regulation (negative)				
252*	*mobI*	151	<0.0001	0.83	0.82	Dtr (auxiliary protein)	homology to MobI			

^a^The 21 ORFs described in Section 3.1 are indicated by asterisks.

^b^Efficiency relative to that of the wild type Rts1.

^c^NT, not tested.

^d^Orf216 contains the TrbC_F type domain and the TraW domain.

^e^Not required for conjugation.

^f^Unknown for their involvement in conjugation.

### 3.2. Identification of the conjugation-related genes in Rts1 by systematic deletion analysis

To experimentally determine the necessity of the 21 genes for conjugation and identify additional genes that could be required for the conjugation of Rts1, we performed a systematic deletion analysis of Rts1 by constructing a series of deletion mutants in which a single gene or a block of genes was deleted in *E. coli*. Finally, we constructed a total of 85 mutants (Fig. 1, Supplementary Table S1), with the largest deletion being a deletion of the 105.4-kb segment encoding *orf7-orf158*. All Rts1 genes, except for the *repA* gene encoding a replication initiation protein, were deleted in the mutants. These mutants were then subjected to a conjugation assay using *E. coli* as the donor and recipient. Note that all mutants were marker-free (see Section 2); thus, it was not necessary to consider the polar effect due to marker gene insertion.

As summarized in Tables 1 and Supplementary Table S1, deletion of 32 genes resulted in significant changes in the conjugation efficiency. The conjugation ability of Rts1 was abolished or severely impaired by the deletion of the abovementioned 21 genes, except for *orf218*, confirming that these genes were required for conjugation. The *orf218* deletion mutant also showed reduced conjugation efficiency, similar to the deletion mutant of the Orf218 homolog in SXT (S063).^19^ The 11 newly identified genes could be divided into three groups according to the transfer phenotypes of their deletion mutants. Deletion of six genes (*orf180, orf186, orf206, orf239, orf247*, and *orf248*) abolished or severely impaired the conjugation ability of Rts1. Of their gene products, two contained domains or motifs indicative of their functions. Orf206 showed homology to thiol oxidoreductase proteins with a thioredoxin fold and the 121Cys-X-X-124Cys motif that can modulate disulfide bonds.^38^ Orf248 contained the SLT domain of lytic transglycosylases. Although a homolog of transglycosylase was also present in SXT, it was not required for the conjugation of SXT.^19^ As mentioned above, the Orf180 homolog of F (TrbE) was not required for the conjugation of F,^37^ in contrast to Orf180, which was essential for the *E. coli*-to-*E. coli* conjugation of Rts1. No significant homologies were found in the remaining three ORFs (Orf186, Orf239, and Orf247).

In contrast to these six genes, deletion of four ORFs (*orf191, orf192, orf223*, and *orf224*) provoked a moderate impairment of conjugation efficiency, ranging from a 1/20 to 1/100 reduction compared with the wild type, similar to the *orf218*-deletion mutant mentioned above (Table 1), indicating that these four ORFs and *orf218* are dispensable but required for the efficient *E. coli*-to-*E. coli* conjugation of Rts1. Orf223 and Orf224 contained RecT and phage_rel_nuc domains, respectively, and Orf224 showed notable homology to S066 of SXT, which was not required for the conjugation of SXT.^19^ No noticeable sequence homology was found for Orf191 and Orf192.

The deletion mutant of the remaining ORF (*orf249*) exhibited a completely different phenotype. This mutant showed remarkably improved conjugation efficiency, a 400-fold improvement compared with the wild type; no significant homology was found for this ORF.

### 3.3. Complementation analysis

To confirm that the products of the 32 genes identified by deletion analysis were actually involved in conjugation, we performed a systematic complementation analysis. From this analysis, we excluded 14 ORFs (*orf201, orf202, orf207-211, orf213, orf216, orf217, orf240-242,* and *orf244*) because their essentiality was apparent from their sequence homologies to known essential genes in the other conjugation systems. In this analysis, we utilized plasmid clones in the pUC18-based Rts1 genomic library, which was used for whole genome random shotgun sequencing and whose end sequences were known.^25^ Specifically, we selected plasmid clones containing an entire target gene (Supplementary Fig. S1) and introduced them into *E. coli* harbouring the corresponding deletion mutant of Rts1. Complementing genes were expressed by the *lacZ* promoter on pUC18 without induction by IPTG. For the complementation of *orf191*, *orf223*, and *orf224*, we used the low copy number plasmid pACYC184 as a vector plasmid (Supplementary Fig. S1 and Table S2) because appropriate pUC18 derivatives were not found (*orf223* and *orf224*) or the introduction of plasmid clone resulted in the inhibition of host cell growth (*orf191*). In the case of *orf223* and *orf224*, a pACYC184 derivative carrying both genes was used.

Of the 18 deletion mutants analysed (Table 1), the conjugation efficiencies of 17 mutants that showed diminished or highly impaired conjugation were improved by introducing complementing plasmid clones, although the efficiencies of several mutants were not restored to the wild-type level. Interestingly, complementation of *orf246*- and *orf247*-deletion mutants resulted in a remarkable improvement in conjugation efficiency, with 1,489- and 97-fold increases compared with wild type, respectively (Table 1). In contrast, complementation of the *orf249*-deletion mutant, which showed an increased conjugation ability, resulted in a 1/40-fold reduction in conjugation efficiency (Table 1). These results suggested that *orf246* and *orf247* positively regulate the gene expression of the Rts1 conjugation system and *orf249* negatively regulates this expression.

### 3.4. Mobilization assay of plasmid pSU4628 in Rts1 mutants

To elucidate the roles of the 32 genes involved in the conjugation process, we conducted a systematic mobilization assay of each deletion mutant using the plasmid pSU4628, an Ap-resistant derivative of the plasmid CloDF13.^28^ pSU4628 is not self-transmissible but is mobilizable if Mpf gene products are provided by a co-resident helper conjugative element, as pSU4628 encodes its own Dtr proteins and T4CP.^28^ In this analysis, two mutants of presumed positive regulator-encoding genes (*orf246* and *orf247*) were unable to mobilize pSU4628, as expected (Table 1). Of the remaining conjugation-deficit mutants, 17 were unable to mobilize pSU4628 (Table 1), indicating that the 17 genes deleted in these mutants are required for Mpf. In addition, four genes (*orf185, orf191, orf206,* and *orf248*) appeared to impact Mpf because their deletion mutants showed very low mobilization. In contrast, eight conjugation-deficit mutants (*orf192, orf201, orf202, orf204, orf218, orf223, orf224* and *orf252*-deletion mutants) were able to mobilize pSU4628 as efficiently as wild-type Rts1 (Table 1). This result indicates that these eight genes are not involved in Mpf. The deletion mutant of *orf249*, which encodes a presumed negative regulator, showed a markedly increased ability to mobilize pSU4628, as expected.

### 3.5. Plasmids with a conjugation system highly homologous to the Rts1 system

**Figure 3.**
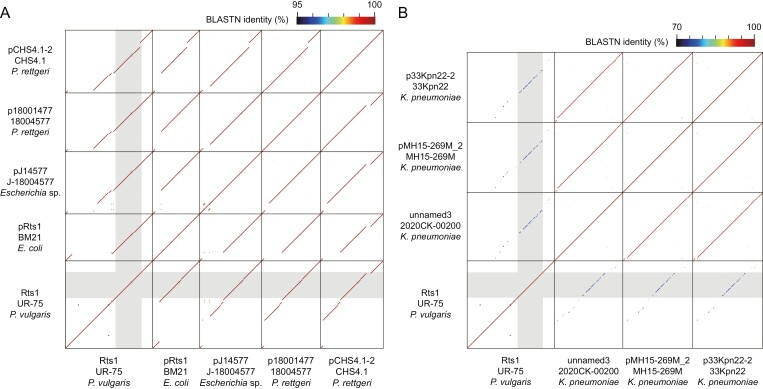
Dot plot matrix analysis of the plasmids with conjugation-related regions nearly identical or similar to that of Rts1. (A) Genomic comparison of Rts1 with six plasmids that possessed a conjugation-related region nearly identical to that of Rts1. (B) Genomic comparison of Rts1 with three plasmids with conjugation-related regions similar to that of Rts1. See Supplementary Fig. S2 for the results of dot plot matrix analysis of all nine plasmids with conjugation-related regions similar to that of Rts1. In both panels, conjugation-related regions are indicated by light grey shading.

**Figure 4.**
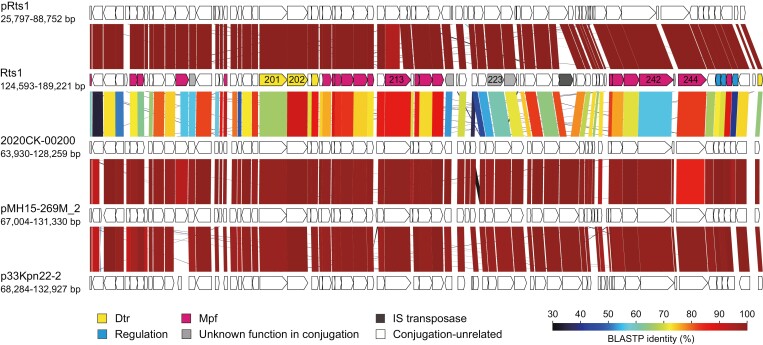
Comparison of conjugation-related regions among Rts1, pRts1, 2020CK-0200, pMH15-269M_2, and p33Kpn22-2. The gene organizations of conjugation-related regions of pRts1, 2020CK-0200, pMH15-269M_2, and p33Kpn22-2 (corresponding to the Rts1 region encompassing *orf180* to *orf252*) and amino acid sequence homologies between genes are shown.

By searching genes similar to Orf201 (relaxase) in Rts1, we identified 14 plasmids that encode conjugation systems almost identical or very similar to that of Rts1 (Table 2). Five plasmids from *Providencia*, *Escherichia*, and *Proteus* species contained a region almost identical to the 64.6-kb region of Rts1 where conjugation-related genes are located (Figs. 3A and 4). Their *rep* genes were also nearly identical (>99.8% nucleotide sequence identity) to that of Rts1 and belonged to the IncT group. However, these plasmids were much smaller than Rts1, and various sizes of regions between the *rep* gene and the conjugation-related region where the Km-resistance gene is located in Rts1 were missing (Fig. 3A). Nine plasmids from *Klebsiella* and *Citrobacter* species also contained a region similar to the conjugation-related region of Rts1 (Fig. 3B, see Supplementary Fig. S2 for the comparison of all members in this group), but the amino acid sequence identities of each gene product were 23–92% of the counterparts of Rts1 (Fig. 4). In addition, only the conjugation-related region showed sequence similarities to Rts1 (Fig. 3B, see also Supplementary Fig. S2), and the Inc type was ‘repA(pKOX)’, as defined by PlasmidFinder. Although many plasmids from various species were found to encode an Orf201 homolog more similar to that of SXT, their amino acid sequence identities to Orf201 were less than 51%. Of note, the homologs of Orf180, which was not present in SXT but essential for the *E. coli*-to-*E. coli* conjugation of Rts1, were present only in the abovementioned two groups of plasmids (Table 2).

**Table 2.** Plasmids encoding proteins highly homologous to Orf201 and Orf180 of Rts1.

Species	Strain	Plasmid	Length (bp)	Orf201		Orf180		Acc. number
				Identity (%)	Coverage (%)	Identity (%)	Coverage (%)	
*Escherichia* sp.	J-18004577	pJ14577	1,54,303	100.0	100.0	100.0	100.0	CP114206.1
*Providencia rettgeri*	18004577	p18001477_Rst	1,46,248	100.0	100.0	100.0	100.0	CP098042.1
*Providencia rettgeri*	CHS4.1	pCHS4.1-2	1,55,985	100.0	100.0	100.0	100.0	OL908907.1
*Providencia rettgeri*	18014577	p18014577_Rst	1,46,248	100.0	100.0	100.0	100.0	MW864328.1
*Escherichia coli*	BM21	pRts1	1,16,713	100.0	100.0	100.0	100.0	MN626604.1
*Klebsiella michiganensis*	2022CK-00495	unnamed3	1,69,137	66.3	100.0	60.3	100.0	CP124763.1
*Klebsiella oxytoca*	2020CK-00213	unnamed1	1,66,629	66.3	100.0	60.3	100.0	CP118213.1
*Klebsiella oxytoca*	2020CK-00200	unnamed3	1,69,765	66.3	100.0	60.3	100.0	CP115682.1
*Citrobacter youngae*	UL-CPE-01	pUL-CPE-01	2,11,078	66.2	100.0	60.3	100.0	CP132280.1
*Klebsiella pneumoniae*	33Kpn22	p33Kpn22-2	1,74,178	66.2	100.0	60.3	100.0	CP069048.1
*Klebsiella oxytoca*	KONIH4	pKOX-ea2b	1,79,680	65.9	100.0	60.3	100.0	CP026273.1
*Klebsiella pneumoniae*	KP-2683-1	pKP-2683-1-174.8	1,74,840	65.9	100.0	62.1	100.0	CP122576.1
*Klebsiella pneumoniae*	BA2105	pKOX	1,78,010	65.9	100.0	60.3	100.0	CP060422.1
*Klebsiella pneumoniae*	MH15-269M	pMH15-269M_2	1,73,855	65.9	100.0	62.1	100.0	AP023339.1

## 4. Discussion

Through a systematic plasmid-wide deletion analysis followed by complementation analyses, we identified a full set of genes required for the efficient conjugation of Rts1 from *E. coli* to *E. coli*, which included 32 genes (Table 1). Of the 32 genes, 23 were essential (conjugation-undetectable or severely impaired for their deletion mutants), and nine were not essential but required for efficient or controlled conjugation (Table 1). Based on the results of mobilization assays along with the phenotypes of deletion mutants and the results of the homology search, genes involved in Mpf and Dtr, the gene encoding the T4CP protein, and the genes involved in regulating the expression of the Rts1 conjugation system were identified, as discussed below. Plasmids with a conjugation system almost identical or very similar to that of Rts1 were also identified by database search (Table 2, Figs. 3 and 4, and Supplementary Fig. S2).

### 4.1. Mpf genes

The results of mobilization assays suggested that 21 genes are involved in Mpf, of which three (*orf186, orf191*, and *orf239*) showed no noticeable homologies to the genes in SXT, R27, and F (Table 1). Of the 21 genes, the essential requirement of *orf180,* which encodes a small protein of 45 amino acids and is conserved in close relatives of Rts1, is interesting because its homolog in plasmid F was not required for conjugation (Table 2 and Fig. 3). The presence of *orf185*, which appears to encode a LepB-like type I signal peptidase (peptidase S26 superfamily; pfam PF00717) with predicted catalytic residues (Ser56 and Lys101) and domains B-E conserved among the type I signal peptidase family, was also interesting.^39^ Rts1 encodes another peptidase, Orf215, which is also required for Mpf and belongs to the TraF protein family with the active residues Lys67 and Asp130.^40,41^ Thus, two peptidases are required for the *E. coli*-to-*E. coli* conjugation of Rts1. To the best of our knowledge, Rts1 is the first conjugative element found to encode more than one conjugation-related protease. In plasmid RP4, TraF processes TrbC, a major component of sex pili, at the C-terminus for its maturation and cyclization, and two host-encoded peptidases, LepB and an unknown peptidase, are necessary for the maturation of TrbC.^42,43^ In Rts1, the pilin protein (Orf196) or some other proteins may require processing by Orf185, but the precise function and substrates of this LepB homolog remain to be elucidated.

Another interesting Mpf gene identified is *orf248,* which is located between the regulator genes and encodes a member of the soluble lytic transglycosylase (SLT) family with three conserved motifs and a presumed catalytic residue, Glu55.^44^ SLTs are widely distributed among type III secretion systems and T4SSs and are thought to be involved in rearrangement of the peptidoglycan layer to form the transport machinery required for penetrating the cell wall.^44^ However, an Orf248 homolog in SXT was not required for conjugation.^19^ R27 also encodes an SLT family protein, but its function has not yet been analysed.^18,34^

The presence of Orf206 was also interesting, as it contains a thioredoxin fold with a catalytic CXXC motif, which implicates it as a thiol disulfide oxidoreductase that catalyses intrachain disulfide bond formation in extracytoplasmic proteins. Many conjugative elements encode one or more thioredoxin-like proteins, and some of their biochemical activities have been experimentally determined.^45–48^ The contribution of these thioredoxin-like proteins to conjugation has also been reported in F, R27, and SXT.^19,45,46^ Although the deletion of the genes for these proteins in F (*trbB*) and R27 (*dsbC*) reduced their conjugation efficiency (10-fold and 10,000-fold reduction, respectively), the reduction was observed only in *dsbC*-null host strains, suggesting the redundancy of host- and plasmid–encoded thioredoxins. In contrast, deletion of *orf206* resulted in a >100-fold decrease in the conjugation efficiency of Rts1, even in the *dsbC*-positive host strain, suggesting that the function of Orf206 is unique and specific to Rts1. A host-encoded *dsbC*-independent phenotype was also observed in SXT, where the deletion of a *dsbC*-homolog (*s054*) abolished its conjugation ability in a *dsbC*-positive *E. coli* strain. Interestingly, S054 is a homolog of Orf212 in Rts1, but Orf212 is not required for the conjugation of Rts1 from *E. coli* to *E. coli*. Although the reason for this difference is unknown, some effect of *orf212* deletion may be observed in the *orf206*-negative background.

### 4.2. Dtr and T4CP genes

Among the eight genes other than Mpf-related genes and regulatory genes, four (*orf201, orf202, orf204*, and *orf252*) were found to be essential for the *E. coli*-to-*E. coli* conjugation of Rts1 (Table 1). Of these, from the results of the homology search, *orf201* and *orf202* apparently encode the relaxase and T4CP of Rts1, respectively. Orf204 contains a domain of unknown function (DUF4400), and Orf252 contains the MobI domain, which has been shown to be responsible for the recognition of the *oriT* locus in IncC plasmids.^49^ Conjugative elements usually encode a couple of auxiliary proteins involved in Dtr.^12^ For instance, TraY and TraM in IncF plasmids and TraH, TraJ, and TraK in IncP plasmids are auxiliary proteins of Dtr that determine the *oriT* specificity or unwinding of the specific locus of the DNA helix to initiate DNA transfer.^12^ According to these well-characterized plasmids, *orf204* and *orf252* presumably play auxiliary functions in DNA metabolism during the conjugation process. Consistent with this, SXT and R27 also contain two auxiliary proteins of Dtr that are essential for their conjugation,^19,33,34^ and Orf204 showed noticeable homology to one of the auxiliary proteins (TraJ) of SXT and R27 (Table 1). Although Orf252 did not show significant sequence similarities to the protein named MobI in SXT and R27, the genes encoding these proteins are directly located next to the *oriT* locus in SXT and R27, as seen for *orf252* of Rts1 (see Fig. 2 for the SXT data).

### 4.3. Regulatory genes

The phenotypes of deletion mutants of three ORFs (Orf246, Orf247, and Orf249) suggested that the *E. coli*-to-*E. coli* conjugation of Rts1 is negatively regulated by Orf249 and positively regulated by Orf246 and Orf247. Although no significant sequence homology was detected between Orf249 and SetR of SXT, which was experimentally identified as a repressor,^31^ Orf249 appears to be a functional homolog of SetR. Furthermore, Orf246 showed homology to SetC, one of the two positive regulators of SXT, and showed homology to FlhC, one of the master regulators of flagella biosynthesis in *E. coli* and *Salmonella*. FlhC forms a hexameric complex with FlhD to work as a transcriptional activator of the class 2 operon, thereby governing the expression of flagellar genes.^50,51^ The *flhC* gene is located just downstream of *flhD* in *E. coli*. In Rts1, *orf246* is also located just downstream of *orf247*. In SXT, *setD*, a homolog of *flhD*, is also located next to *setC* (Fig. 2), and SetC and SetD were experimentally shown to work as transcriptional activators by binding to several promoters of conjugation genes.^52^ In addition, IncA/C plasmids encode two conjugation-activating genes, *acaC* and *acaD,* which are homologous to *flhC* and *flhD*, respectively, and AcaC and AcaD are copurified, suggesting that they form a heteromeric complex.^53–55^ Considering these observations, Orf246 and Orf247 likely form a heteromeric complex to work as activators.

### 4.4. Other genes required for the efficient *E. coli*-to-*E. coli* conjugation of Rts1

The roles of the remaining four gene products (Orf192, Orf218, Orf223, and Orf224) are currently unknown, but the conjugation efficiencies of their deletion mutants were 1/10–1/100 of that of wild-type Rts1 (Table 1). Of these, Orf223 and Orf224 contained the RecT and phage_rel_nuc domains, respectively. Interestingly, S065 and S066 of SXT also contained these domains, and the latter showed noticeable homology to Orf224. These SXT genes were named *bet* and *exo* based on the homologies to the *bet* and *exo* genes of lambda phage and experimentally shown to have RecA-independent homologous recombination activity similar to the lambda system.^56,57^ However, in contrast to Rts1, these genes were not required for the conjugation of SXT.^19^ Orf218 also showed sequence similarity to S063 of SXT, and the *s063*-deletion mutant and the *orf218*-deletion mutant both showed a reduction in conjugation efficiency^19^ (Table 1), but the functions of S063 are also currently unknown. The function of *orf192* also remains to be investigated, as no significant sequence homology to known functionally characterized genes was found.

## Supplementary Material

dsae002_suppl_Supplementary_Tables_S1-S3

dsae002_suppl_Supplementary_Figures_S1-S2
