# Supplementary material for: A comprehensive list of genes required for the efficient conjugation of plasmid Rts1 was determined by systematic deletion analysis
Source: DNA Res. 2024 Feb 1;31(1):dsae002. doi: 10.1093/dnares/dsae002 (PMC10838148; doi:10.1093/dnares/dsae002)
Supplement: dsae002_suppl_Supplementary_Figures_S1-S2 [file dsae002_suppl_supplementary_figures_s1-s2.zip › dsae002_suppl_Supplementary_Figures_S1-S2/dsae002_suppl_Supplementary_Figures_S1-S2.docx]

**Supplementary data**

**Supplementary Figure S1.** The gene organization of Rts1 and the DNA fragments used for complementation analysis.

The DNA regions carried by each plasmid clone are indicated by lines. The plasmid pUC18 was used as a vector for complementation analysis except for two fragments, for which pACYC184 was used as a vector (indicated by asterisks). The conjugation-related genes that were identified in this study are indicated in gray. IS*2A* is indicated by a black rectangle.

**Supplementary Figure S2.** Dot plot matrix analysis of the nine plasmids with conjugation-related regions similar to that of Rts1.

(A) Comparison of Rts1 with the nine plasmids. (B) Comparison between the nine plasmids. Conjugation-related regions are indicated by light gray shading.

**Supplementary Table S1.** Conjugation efficiencies of Rts1 mutants and primers used for constructing each mutant.

**Supplementary Table S2.** Primers used for the construction of pACYC184-based complementation vectors.

**Supplementary Table S3.** Relationships and differences between the CDS annotations of SXT that were used in this study (Dr. Burrus; personal communication) and those that were deposited in NCBI (accession no. DS990138.1).
