# Supplementary figures and images for: A comprehensive list of genes required for the efficient conjugation of plasmid Rts1 was determined by systematic deletion analysis
Source: DNA Res. 2024 Feb 1;31(1):dsae002. doi: 10.1093/dnares/dsae002 (PMC10838148; doi:10.1093/dnares/dsae002)

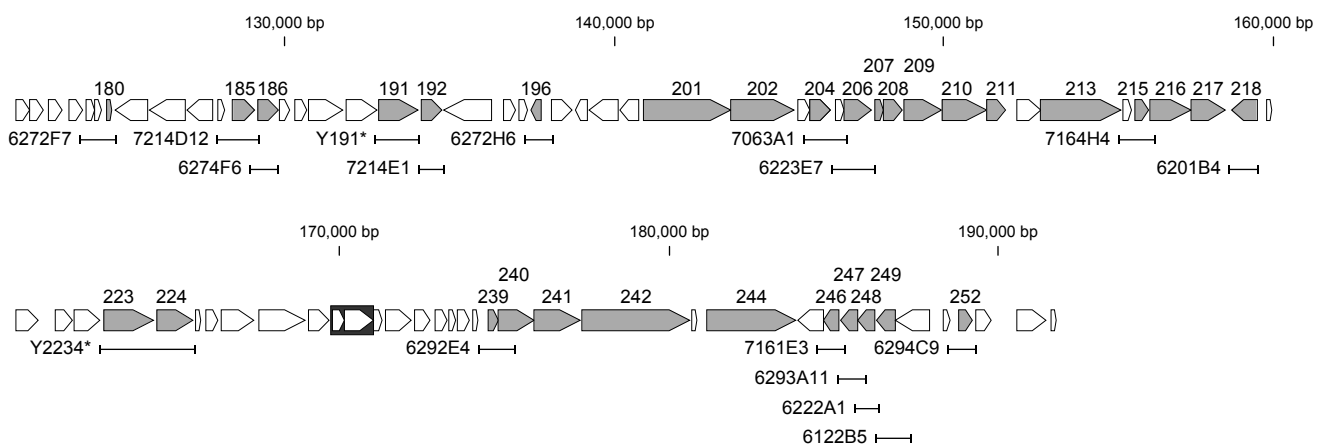

Supplementary Fig. S1.

A

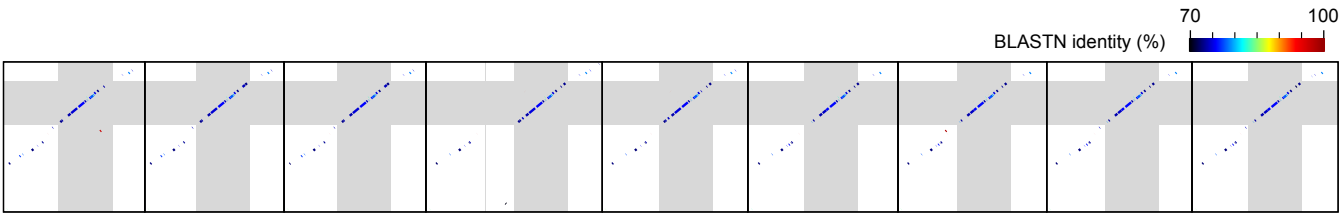

B

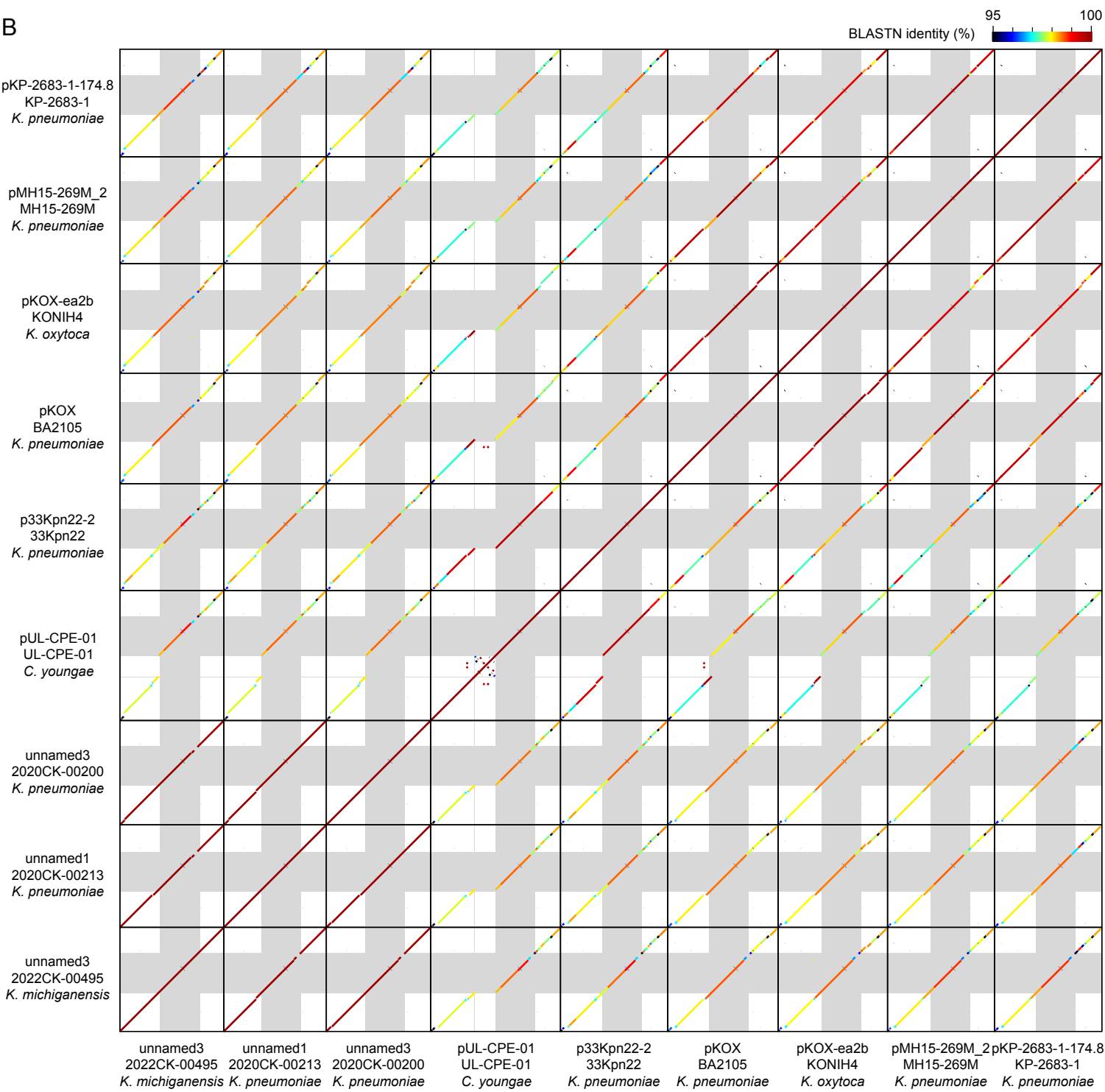

Supplementary Fig. S2.

Supplement: dsae002_suppl_Supplementary_Figures_S1-S2 [file dsae002_suppl_supplementary_figures_s1-s2.zip › dsae002_suppl_Supplementary_Figures_S1-S2/dsae002_suppl_Supplementary_Figures_S1-S2.pdf]
